# RNA‐Seq‐based discovery of genetic variants and allele‐specific expression of two layer lines and broiler chicken

**DOI:** 10.1111/eva.13557

**Published:** 2023-05-22

**Authors:** Muhammad Arsalan Iqbal, Frieder Hadlich, Henry Reyer, Michael Oster, Nares Trakooljul, Eduard Murani, Alvaro Perdomo‐Sabogal, Klaus Wimmers, Siriluck Ponsuksili

**Affiliations:** ^1^ Research Institute for Farm Animal Biology Institute of Genome Biology Dummerstorf Germany; ^2^ Faculty of Agricultural and Environmental Sciences University Rostock Rostock Germany

**Keywords:** allele‐specific expression, broiler, fixation index, laying hen, RNA‐seq, SNPs

## Abstract

Recent advances in the selective breeding of broilers and layers have made poultry production one of the fastest‐growing industries. In this study, a transcriptome variant calling approach from RNA‐seq data was used to determine population diversity between broilers and layers. In total, 200 individuals were analyzed from three different chicken populations (Lohmann Brown (LB), *n* = 90), Lohmann Selected Leghorn (LSL, *n* = 89), and Broiler (BR, *n* = 21). The raw RNA‐sequencing reads were pre‐processed, quality control checked, mapped to the reference genome, and made compatible with Genome Analysis ToolKit for variant detection. Subsequently, pairwise fixation index (*F*_ST_) analysis was performed between broilers and layers. Numerous candidate genes were identified, that were associated with growth, development, metabolism, immunity, and other economically significant traits. Finally, allele‐specific expression (ASE) analysis was performed in the gut mucosa of LB and LSL strains at 10, 16, 24, 30, and 60 weeks of age. At different ages, the two‐layer strains showed significantly different allele‐specific expressions in the gut mucosa, and changes in allelic imbalance were observed across the entire lifespan. Most ASE genes are involved in energy metabolism, including sirtuin signaling pathways, oxidative phosphorylation, and mitochondrial dysfunction. A high number of ASE genes were found during the peak of laying, which were particularly enriched in cholesterol biosynthesis. These findings indicate that genetic architecture as well as biological processes driving particular demands relate to metabolic and nutritional requirements during the laying period shape allelic heterogeneity. These processes are considerably affected by breeding and management, whereby elucidating allele‐specific gene regulation is an essential step towards deciphering the genotype to phenotype map or functional diversity between the chicken populations. Additionally, we observed that several genes showing significant allelic imbalance also colocalized with the top 1% of genes identified by the F_ST_ approach, suggesting a fixation of genes in cis‐regulatory elements.

## 1 INTRODUCTION

Poultry farming is one of the fastest‐growing industries, and it is expected to continue growing as human population growth drives demand for meat and eggs (Mottet & Tempio, 2017; Talebi et al., 2020). Traditionally, the domestic chicken populations were bred for two main reasons egg and meat production (Rubin et al., 2010). Over 120 million tons of meat and 1.2 trillion eggs are consumed globally each year from commercial broiler and layer suppliers. For this reason, the poultry industry keeps more than 70 billion chickens yearly to fulfill consumer demand (Qanbari et al., 2019). The selection progress in layer chicken was mainly driven by commercial egg production and efficient feed conversion, whereas in broilers, the emphasis was on rapid growth, that is, achieving a 50–60 times increase in body weight (BW) from hatch to market. This selective breeding has resulted in considerable advancements specifically for individual traits (Havenstein et al., 2003).

In comparison with other commercial broiler strains around the globe, the Cobb 500 broiler has a high growth performance and comparable breast meat production at different processing ages (Coneglian et al., 2010; Taschetto et al., 2012). The parents and grandparents of the Cobb 500 broiler belong to Cobb‐Vantress Inc., which was established in 1916 and is one of the oldest broiler breeding companies in the world (www.cobb‐vantress.com). Over the past 50 years, LSL and LB layers have established themselves as the world's leading commercial laying lines. These lines have been specifically selected for their performance in egg production and carefully selected for their combination of capabilities (http://www.ltz.de/en/layers/). Interestingly, both lines show similar egg production performance, but genetic differences between the two lines are responsible for the physiological differences, which in turn has led to variations in certain traits such as body weight, immunity, bone metabolism, phytate degradation, and transcription abundance of genes that could be attributed to immune system processes and phosphorus metabolism (Habig et al., 2012; Iqbal et al., 2022; Reyer et al., 2021; Schmucker et al., 2021; Sommerfeld, Huber, et al., 2020; Sommerfeld, Omotoso, et al., 2020).

Many studies suggested that the difference in growth performance between broilers and layers was mainly due to the higher feed intake in broilers (Hocking et al., 1997; Mahagna & Nir, 1996; Masic et al., 1974). Nevertheless, several recent studies compared layers to broilers and highlighted numerous breed‐specific features, including growth‐rate performance, physiological traits, metabolism, and immunological functions (Koenen et al., 2002; Nihashi et al., 2019; Qanbari et al., 2019; Talebi et al., 2020). One study revealed that broilers at 6 weeks of age have a 5‐fold greater BW than layers, with most of the difference in BW attributed to increased growth in broilers during the first 2 weeks after hatch (Zhao et al., 2004). Scheuermann et al. (2004) found that the higher BW of broilers was primarily due to the size of skeletal muscles, as broiler muscles have more myofibers with larger diameters than layers. Comparison of liver function in broilers and layer chickens at various stages of embryonic development and hatch indicates that on day 18 of embryogenesis, broiler liver triacylglycerol level is 1.3 times higher in broilers than in layer, and 2.2 fold higher at chick hatch (Buzała et al., 2015; Sato et al., 2006). Comparison of inflammatory responses to lipopolysaccharide (LPS) revealed that both breeds showed differences in cytokine expression and immune responses with the broiler chicken suppressing the inflammatory responses, indicating a positive correlation with growth rate (Leshchinsky & Klasing, 2001). The egg production performance of the two‐layer strains LB and LSL is almost comparable, but they differ significantly in phenotypic traits, immunological function, gene expression, and metabolic activities. A multi‐omics study discovered that the LB and LSL layer lines use divergent intrinsic mechanisms that shape their immune and metabolic function (Iqbal et al., 2022). Another pan‐omics longitudinal integration study at different production periods from pre‐laying to the onset of laying indicated that they differed in their ability to activate immune and metabolic mechanisms, as well as unique gut‐microbiota interactions (Ponsuksili et al., 2022).

Recently several studies demonstrated that computational approaches to identify variants and accurate mapping of RNA‐seq reads is an effective and cost‐efficient source for the detection of genomic variations (Adetunji et al., 2019; Cornwell et al., 2018; Jehl et al., 2021; Piskol et al., 2013; Tang et al., 2014). Previous studies have shown that nonsynonymous, synonymous, and noncoding SNPs derived from RNA‐seq data can represent markers of genetic differentiation, particularly when they are targets for selection or involved in the regulation of gene expression (Page & Chapman, 2021; Sun et al., 2019). Allele‐specific expression (ASE) is a phenomenon involving unbalanced expression results in a selection process in which one allele is preferentially expressed over another, with the potential functional consequence on phenotype (Pierre et al., 2022).

In this study, we used transcriptomic data to identify SNPs and short indels in expressed genes to investigate genetic variation in three different chicken populations. F_ST_ screening was used to identify regions of genetic differentiation resulting from the domestication of chickens and subsequent specialization into broiler and layer lines. Focusing on SNPs detected in expressed regions, characterized variants affecting protein functions to study cis‐regulated genes by analyzing allele‐specific expression in the jejunum mucosa of LB and LSL strains at different time points (10, 16, 24, 30, and 60 weeks).

## 2 MATERIALS AND METHODS

### 2.1 RNA‐Seq data collection and preprocessing

This study used previously published RNA sequencing data that were deposited by us in the ArrayExpress database at EMBL‐EBI under accession numbers: E‐MTAB‐9137 and E‐MTAB‐9109 for Lohmann Brown (LB) and Lohmann Selected Leghorn (LSL) laying hens. In addition, RNA sequencing data from the broiler (BR, Cobb500; ArrayExpress database at EMBL‐EBI under accession numbers: E‐MTAB‐6169 and E‐MTAB‐12147), were used. Information on sampling and experimental design for the two‐layer strains is documented in Omotoso et al. (2021) and Reyer et al. (2021).

Briefly, the broiler chicken population comprised mixed‐sex Cobb 500 birds from two experiments (trial 1, *n* = 13; trial 2, *n* = 8) described previously in Metzler‐Zebeli et al. (2018) and Reyer et al. (2018). RNA extraction and RNA‐seq were performed from the breast, duodenum, ileum, and jejunum in trial 1 and from muscle and liver samples in trial 2. RNA‐seq reads from tissues were combined and analyzed for variant discovery. A total of 200 chickens were selected from two‐layer lines (Lohmann Brown [LB], *n* = 90; Lohmann Selected Leghorn [LSL], *n* = 89; Broiler [BR], *n* = 21). In the initial preprocessing step, all raw sequencing reads from LB, LSL, and BR were examined for quality control and trimming approach using FastQC (version 0.11.7). The information on the average number of reads/library or strains that were included in the analysis is shown in Table S1.

### 2.2 Mapping and variant detection

Sequenced reads were processed in accordance with GATK (version 4.2.0.0) best practices for reliable SNP detection and genotype calling in RNAseq (Jehl et al., 2021). Initially, we used STAR (version 2.7.8a; Dobin et al., 2013) in 2‐pass mode for mapping to the Ensembl *Gallus gallus* reference genome assembly (GRCg6a, with annotation version 104). In resulting BAM files, multiple alignments are rejected using samtools (version 1.12; Danecek et al., 2021). Further data cleanup steps included removing duplicate reads, splitting reads by cigar string to detect splicing events, and base recalibration to known variants from Ensembl v94's dbSNP (Hunt et al., 2018). Subsequently, discovered SNP and indel variants were counted for each individual using GATK HaplotypeCaller using options for minimum confidence threshold = 20 and to avoid soft‐clipped bases.

Finally, all individual gVCF files were combined and genotyped with a minimum phred‐scaled confidence threshold = 20 (McKenna et al., 2010; Van der Auwera et al., 2013). The created VCF file was subjected to SNP variants selection and filtration with filters FisherStrand >30 and QualByDepth <2 to obtain SNPs for each individual. The numeric recording of genotypes was performed using the “Recode Genotypes” method within the JMP Genomics v.10.1 (SAS Institute).

Two individuals from the LB strain were excluded from the analysis after GATK SNP identification. One animal had a low SNP density, while the other animal did not cluster with other individuals from LB strain as confirmed with Bioconductor R package arrayQualityMetrics (version 3.52.0).

### 2.3 Fixation index (F_ST_ ) estimation and candidate gene selection

Initially, the fixation index (*F*_ST_) for each SNP was calculated between the three different chicken breed groups (LB vs. LSL, LB vs. BR, and LSL vs. BR) by using Weir and Cockerham‐based approach within the SNPRelate R package (version 1.24.0; Weir & Cockerham, 1984; Zheng et al., 2012). During the *F*_ST_ calculation, SNPs on nonautosomes or monomorphic were excluded. Only autosomal SNPs with minor allele frequency (MAF ≥ 0.05) were shortlisted.

Subsequently, the “aggregate” function within the R programming environment was used for the gene‐based aggregation of SNP‐specific *F*_ST_, employing the cutoff criteria to exclude genes represented by <3 SNPs. *Z*‐transformations were performed on gene‐specific *F*_ST_ values according to the following formula: [*Z* (*F*_ST_) = (*F*_ST_ − μF_ST_)/σF_ST_], in this equation, the overall mean of *F*_ST_ values were indicated by (μF_ST_), while the standard deviation was symbolized by (σF_ST_). For visualization, the manhattan plot of the *Z* (*F*_ST_) value for each gene was plotted using CMplot Package Version 4.1.0. During the screening, genes with a *Z* (*F*_ST_) ≥ 4 were selected as candidates. Principle Component Analysis (PCA) was performed with the “snpgdsPCA” function of the SNPRelate.

### 2.4 KEGG and IPA pathway analyses

Only the genes having the top 1% of *Z* (*F*_ST_) values from all the three comparisons: (LB vs. LSL, LB vs. BR, and LSL vs. BR) were subjected to ClueGO (version 2.5.1) and Cluepedia (version 1.5.7) plugin in Cytoscape (version.3.6.1) environment (Bindea et al., 2009, 2013; Shannon et al., 2003). In ClueGO, each comparison was considered a cluster, and right‐sided hypergeometric tests were performed to calculate the *p*‐value. Benjamin–Hochberg was used for multiple testing corrections and the genome of *Gallus gallus* was selected as a reference. KEGG pathways with *p* < 0.05 were considered significant. For ASE genes for each group of laying strain and age were subjected to pathway analysis using the Ingenuity Pathway Analysis (IPA) software (Qiagen). IPA categorizes genes based on annotated gene functions and statistically tests for the representation of functional terms within the gene list and then calculates adjusted *p*‐values using the Benjamini–Hochberg approach.

### 2.5 Variant effect predictor (VEP) analysis

The Ensemble variant effect prediction (VEP) tool (version 106) was used to annotate 282,587 SNPs for the species *Gallus gallus*. Siftb was used to determine both terms (tolerated and deleterious) and also to calculate the SIFT score (0 to 1) based on sequence homology and amino acid properties (McLaren et al., 2016). Qualitative predictions were derived from the SIFT score, such that substitutions with a score of 0.05 were termed “deleterious” and all others were considered “tolerated”.

### 2.6 Allele‐Specific expression (ASE) analysis

To obtain allele‐specific expression (ASE) for SNP variants, the GATK best practice pipeline was run a second time. Discovered SNPs from the first round (explained in Section 2.2) were N‐masked in the reference genome using bedtools (version v2.27.1) and were used for mapping to obtain unbiased STAR alignments. Next, performing residual GATK best practice steps generated unbiased ASE counts. Only results of SNP variants that were initially masked in the reference were retained. A minor allele count of 3 or more reads, total SNP count of 30 or more, and minor allele ratio of a minimum of 1% were required per accepted SNP and sample.

Allele‐specific expression (ASE)‐based RNA sequencing detection was performed within LB and LSL laying strains at each age of laying hens (10, 16, 24, 30, and 60 weeks) using ASEP (Allele‐Specific Expression Analysis in a Population, version 0.1.0) in the R programming environment (Fan et al., 2020). The concept of the “ASE_detection” function from the ASEP package was applied to detect significant (*p*‐value <0.05) gene‐level ASE effects within the population of each age and each strain combination. Initially, the parameters set for the ASE‐detection were unphased, adaptive, and resample rate of 1e6.

Finally, ASE results for all 10 individuals per line and time point were analyzed, to identify strain‐specific genes with ASE, as well as genes with ASE that were shared between weeks within strains and genes with ASE common between strains across time points by using UpSetR (version 1.4.0) (Conway et al., 2017). Statistical significance was considered for genes showing allele‐specific expression with a *p*‐value of <0.05.

## 3 RESULTS

An RNA sequencing‐based approach was used to discover variants in three chicken breeds, LB, LSL, and BR. Initially, 200 individuals [LB (*n* = 90), LSL (*n* = 89), and BR (*n* = 21)] were used for variant detection analysis. With the Genome Analysis ToolKit (GATK) pipeline for variant detection, over five million (5,874,467) variants were detected from three chicken populations: LB 4,232,069, LSL 4,296,844, and BR 4,890,677.

Overall, 80% of individuals shared 282,587 SNPs that were processed for pairwise *F*_ST_ analysis. Additionally, a total of 292,485 commonly identified SNPs in the two‐layer strains were analyzed to determine Allele‐Specific Expression (ASE) within LB and LSL layer lines at each week's age (10, 16, 24, 30, and 60).

### 3.1 Genetic differentiation of chicken breeds based on SNP genotype data

**FIGURE 1:**
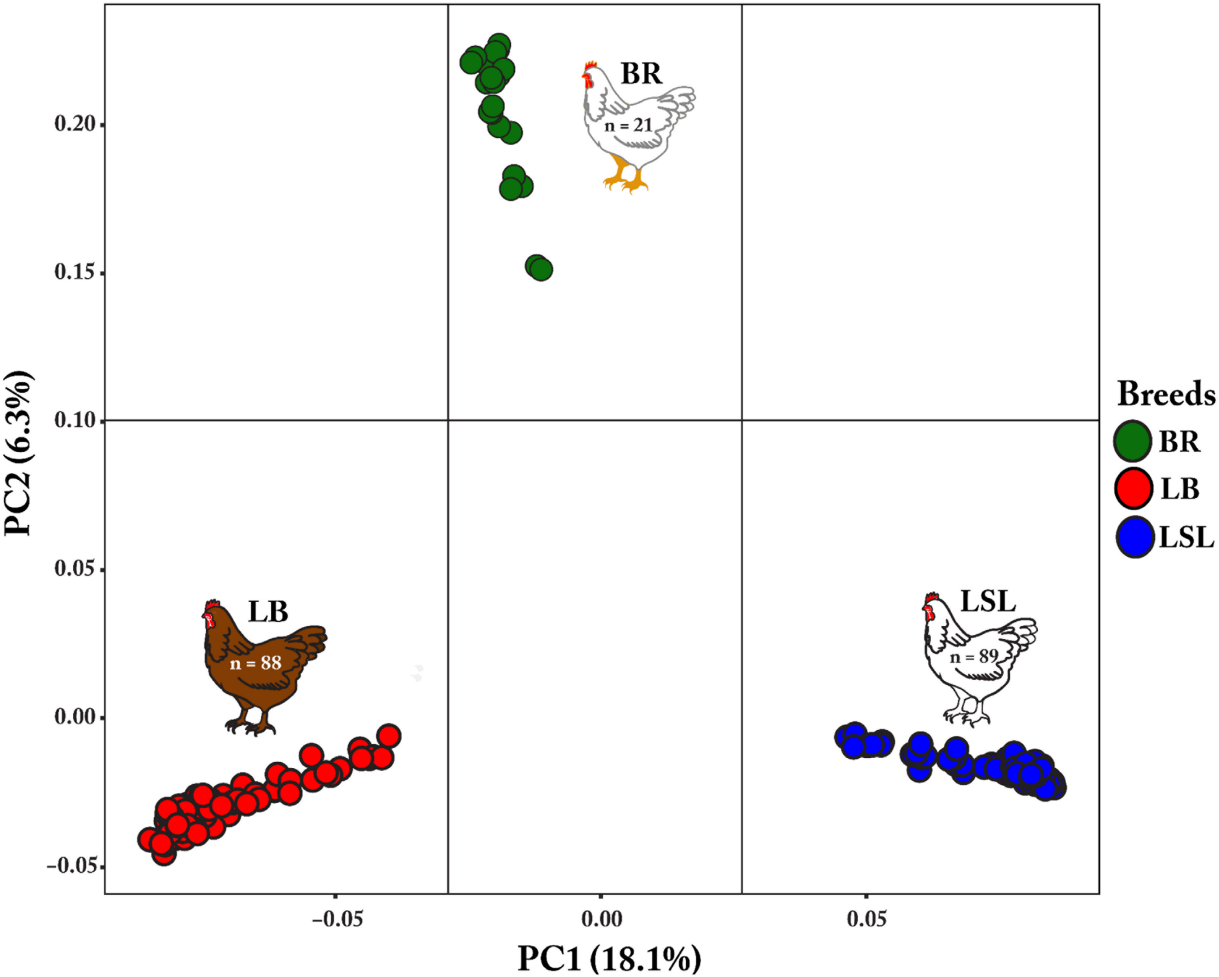
Principle Component Analysis (PCA) of three chicken breeds. PCA plot demonstrates the breed‐specific clustering based on SNP genotype data. Individuals were colored based on their breed; the red circle represents LB, the blue circle indicates LSL, and the green circle depicts BR.

The breed structure was determined by using principal component analysis based on SNP genotype data. There was a strong separation between all three breeds with tight clustering within LB, LSL, and BR breeds revealing high levels of similarity among the individuals within the breed (Figure 1). As expected, noticeable separation among the breeds was observed due to their genetic diversity.

### 3.2 SNPs annotation and SIFT prediction

A total of 282,587 SNPs were processed with Ensembl Variant Effect Predictor (VEP) tool, and annotation analysis of variants placed the identified SNPs in different genomic regions. Out of 282,587 variants, the majority (86,546, 30.63%) were located within predicated the coding sequence. SNPs within coding regions were classified as synonymous and nonsynonymous variants, while nonsynonymous variants were further classified as either missense or nonsense.

**FIGURE 2:**
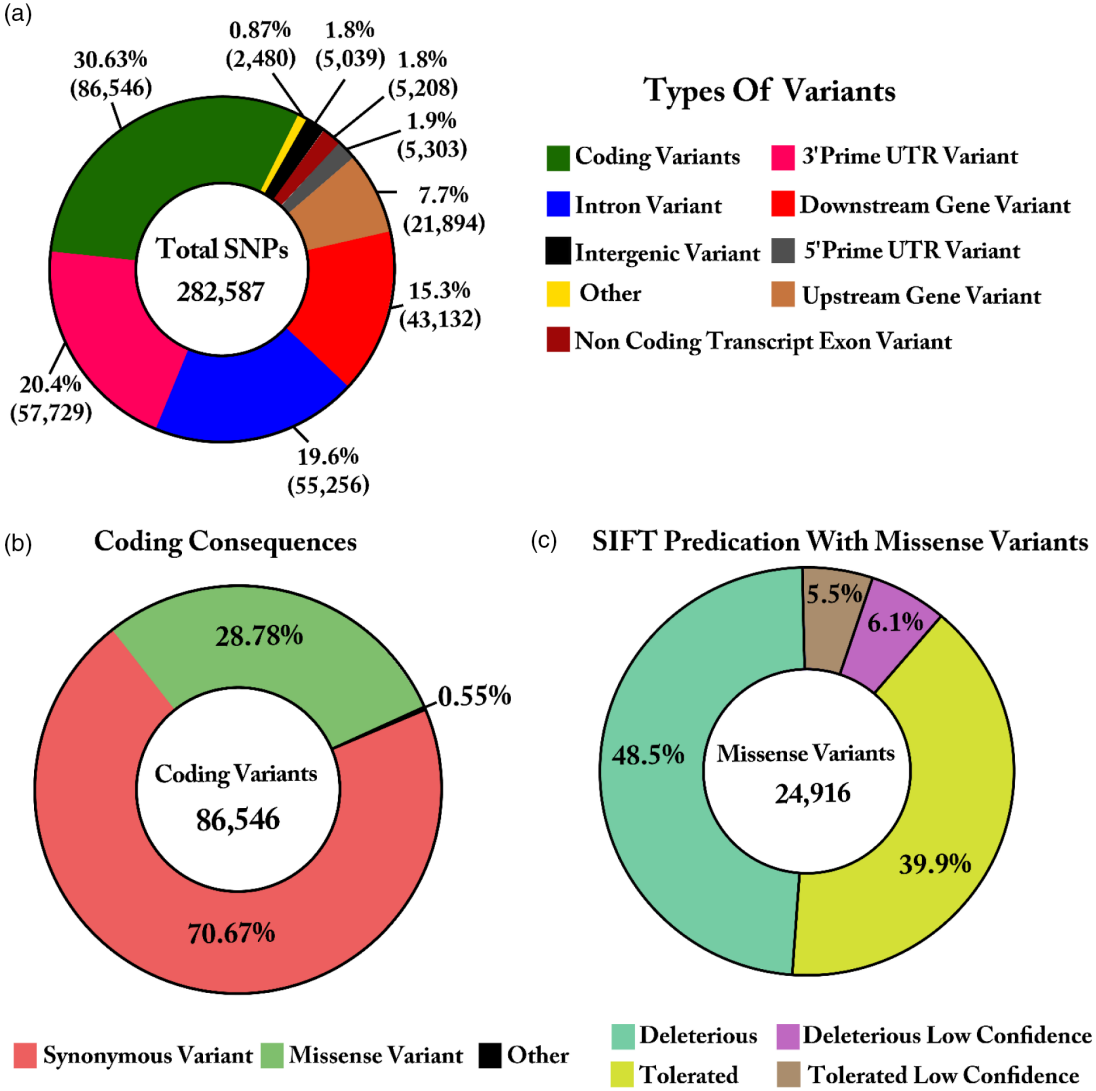
Distribution of SNPs in different genomic regions and prediction of SIFT. (a) The donut chart represents the proportion of SNPs distributed in different genomic regions. In total, 282,587 SNPs were allocated in different regions of the genome which are separated with different colors. (b) The donut plot indicates 86,546 variants in coding sequences. (c) The donut chart illustrates the proportion of missense variants that were considered as tolerated or deleterious and tolerated low confidence or deleterious low confidence based on their SIFT score.

The second most prominent category was variants in the 3'prime UTR regions (57,729; 20.4%) followed by 55,256 (19.6%) and 43,132 (15.3%) variants in introns and downstream gene regions, respectively. As the study was based on RNA sequencing, the SNPs detected in the intronic regions might be immature transcripts. Other variants detected were assigned to the upstream gene regions (21,894; 7.7%), 5'prime UTR (5303; 1.9%), noncoding transcript exons (5208; 1.8%), and intergenic regions (5039; 1.8%; Figure 2a). Of the 86,546 SNPs in the coding sequences, 70.67% were synonymous variants, 28.78% were missense variants, and only 0.55% were other variants, including stop‐gained, stop‐lost, start‐lost, and stop‐retained variants as shown in Figure 2b.

Based on the fact that missense variants affect protein‐coding sequences, these 24,916 variants were used for SIFT prediction analysis. According to our findings, 48.5% of SNPs were predicted to have deleterious effects (0.0–0.05), 39.9% had tolerated effects (0.05–1.0), while 6.1% and 5.5% of SNPs were classified as deleterious low confidence, and tolerated low confidence, respectively (Figure 2c).

### 3.3 Pairwise F_ST_ analysis to detect differentiated genomic regions between LB and LSL

In the pairwise *F*_ST_ analysis between LB and LSL, a total of 282,587 SNPs were processed. As part of the filtering process, 7490 nonautosomal SNPs were excluded along with 72,754 monomorphic or low‐frequency (MAF < 0.05) SNPs. Subsequently, 202,343 SNPs were used for gene‐based aggregation of SNP‐specific F_ST_ values.

**FIGURE 3:**
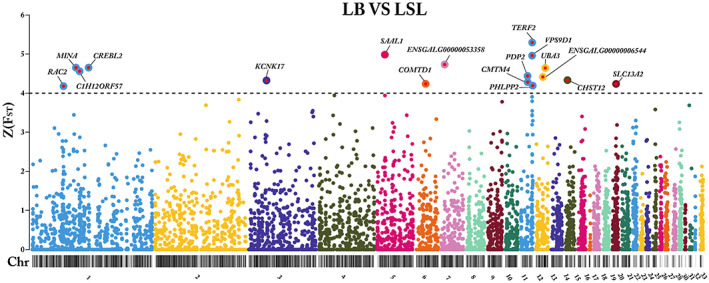
Detection of genetic differentiation between LB and LSL. Manhattan plot of gene‐specific *Z* (*F*_ST_) values comparing LB and LSL layer strains. The black ideogram on the x‐axis shows the chromosomes, whereas the y‐axis indicates the *Z* (*F*_ST_) score for each gene. The black line indicates the cutoff threshold and candidate genes that met the cutoff criterion of *Z* (*F*_ST_) ≥ 4 are marked with gene symbols.

A total of 8929 genes represented by more than or equal to three SNPs each were indicated to differentiate between LB and LSL. Of these, 17 genes passed the cutoff criteria of *Z* (*F*_ST_) ≥ 4 and were considered candidate genes. These candidate genes were distributed on the following chromosomes: 1, 3, 5, 6, 7, 11, 12, 14, and 19. Our results revealed that 5 out of 17 genes, including Telomeric Repeat Binding Factor 2 (*TERF2*), VPS9 Domain Containing 1 (*VPS9D1*), Pyruvate Dehydrogenase Phosphatase Catalytic Subunit 2 (*PDP2*), CKLF Like MARVEL Transmembrane Domain Containing 4 (*CMTM4*), and PH Domain And Leucine Rich Repeat Protein Phosphatase 2 (*PHLPP2*) showed the strongest peak on chromosome 11 with *Z* (*F*_ST_) values ranging from 4.19 to 5.29. Furthermore, 4 genes including CAMP Responsive Element‐Binding Protein Like 2 (*CREBL2*), MYC‐Induced Nuclear Antigen (*MINA*), Chromosome 1 Open Reading Frame 57 (*C1H12ORF57*), and Rac Family Small GTPase 2 (*RAC2*) were located on chromosome 1 with *Z* (*F*_ST_) values ranging from 4.17 to 4.65 (Figure 3).

The other 8 genes comprise Potassium Two Pore Domain Channel Subfamily K Member 17 *(KCNK17)* [*Z* (*F*_ST_) = 4.32] on chromosome 3, Serum Amyloid A Like 1 (*SAAL1*) [*Z* (*F*_ST_) = 4.98] on chromosome 5, Catechol‐O‐Methyltransferase Domain Containing 1 (*COMTD1*) [*Z* (*F*_ST_) = 4.23] on chromosome 6, *ENSGALG00000053358* [*Z* (*F*_ST_) = 4.73] on chromosome 7, Ubiquitin Like Modifier Activating Enzyme 3 (*UBA3*) [*Z* (*F*_ST_) = 4.64] and *ENSGALG00000006544* [*Z* (*F*_ST_) = 4.41] on chromosome 12, Carbohydrate Sulfotransferase 12 (*CHST12*) [*Z* (*F*_ST_) = 4.33] on chromosome 14, and Solute Carrier Family 13 Member 2 (*SLC13A2*) [*Z* (*F*_ST_) = 4.23] on chromosome 19, as shown in Figure 3.

### 3.4 Pairwise *F*_ST_ analysis to detect differentiated genomic regions between LB and BR

In the pairwise *F*_ST_ analysis for the comparison between LB and BR, a total of 282,587 SNPs were processed. As part of the filtering process, 7490 nonautosomal SNPs and 40,824 monomorphic or low‐frequency SNPs (MAF < 0.05) were excluded. Subsequently, 234,273 SNPs were used for gene‐based aggregation of SNP‐specific *F*_ST_ values.

A total of 9056 genes represented by more than or equal to three SNPs were indicated to differentiate between LB and LSL. Of these, 16 genes passed the cutoff criteria of *Z* (*F*_ST_) ≥ 4. These candidate genes were distributed on chromosomes: 3, 4, 5, 6, 7, 11, 15, 20, 24, 25, 27, 31, and 33, respectively. Our results revealed that the gene alpha‐1,2‐mannosyltransferase (*ALG9*) located on chromosome 24 showed the highest *Z* (*F*_ST_) value of 6.28. The genes on chromosome 7 indicate the second highest *Z* (*F*_ST_) values (Shugoshin 2 [*SGO2*], *Z* (*F*_ST_) = 5.09 and HSPB1‐Associated Protein 1 [*HSPBAP1*], *Z* (*F*_ST_) = 4.62) followed by genes on chromosome 25 (S100 Calcium‐Binding Protein A16 [*S100A16*], *Z* (*F*_ST_) = 4.62, and Interleukin 6 Receptor [*IL6R*], *Z* (*F*_ST_) = 4.45).

**FIGURE 4:**
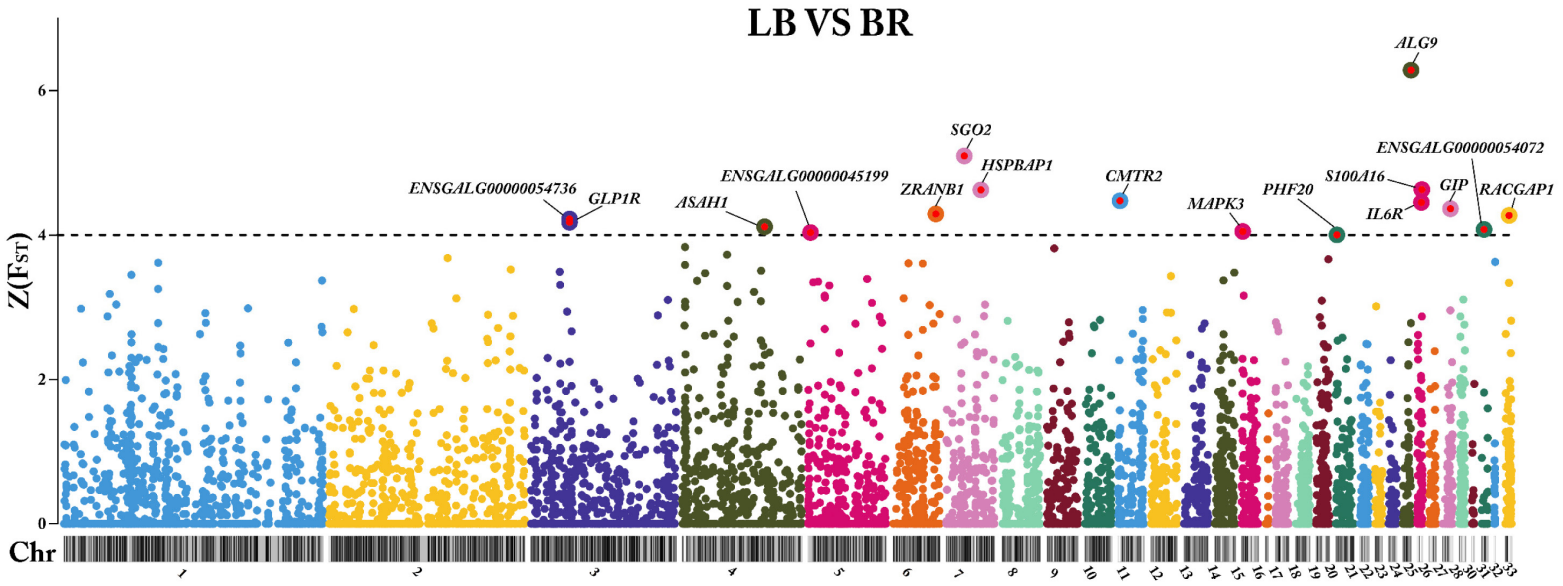
Detection of genetic differentiation between LB and BR. Manhattan plot of gene‐specific *Z* (*F*_ST_) values comparing LB and BR breeds. The black ideogram on the x‐axis shows the chromosomes, whereas the y‐axis indicates the *Z* (*F*_ST_) score for each gene. The black line indicates the cutoff threshold and candidate genes that met the cutoff criterion of *Z* (*F*_ST_) ≥ 4 are marked with gene symbols.

The other 11 candidate genes, including *ENSGALG00000054736* (*Z* (*F*_ST_) = 4.22) and *GLP1R* (*Z* (*F*_ST_) = 4.17) on chromosome 3, N‐Acylsphingosine Amidohydrolase 1 (*ASAH1*) (*Z* (*F*_ST_) = 4.11) on chromosome 4, *ENSGALG00000045199* (*Z* (*F*_ST_) = 4.03) on chromosome 5, Zinc Finger RANBP2‐Type Containing 1 (*ZRANB1*) (*Z* (*F*_ST_) = 4.29) on chromosome 6, Cap Methyltransferase 2 (*CMTR2*) (*Z* (*F*_ST_) = 4.47) on chromosome 11, Mitogen‐Activated Protein Kinase 3 (*MAPK3*) (*Z* (*F*_ST_) = 4.04) on chromosome 15 PHD Finger Protein 20 (*PHF20*) (*Z* (*F*_ST_) = 4.0) on chromosome 20, Gastric Inhibitory Polypeptide (*GIP*), (*Z* (*F*_ST_) = 4.36) on chromosome 27, *ENSGALG00000054072* (*Z* (*F*_ST_) = 4.07) on chromosome 31, and Rac GTPase‐Activating Protein 1 (*RACGAP1*) (*Z* (*F*_ST_) = 4.27) on chromosome 33, as shown in Figure 4.

### 3.5 Pairwise *F*_ST_ analysis to detect selection signature between LSL and BR

In the pairwise F_ST_ analysis for the comparison between LSL and BR, a total of 282,587 SNPs were processed. Hence, 7490 non‐autosomal SNPs and 53,654 SNPs that were monomorphic or had a MAF of <0.05 were filtered out. Subsequently, 221,443 SNPs were used for gene‐based aggregation of SNP‐specific F_ST_ values.

**FIGURE 5:**
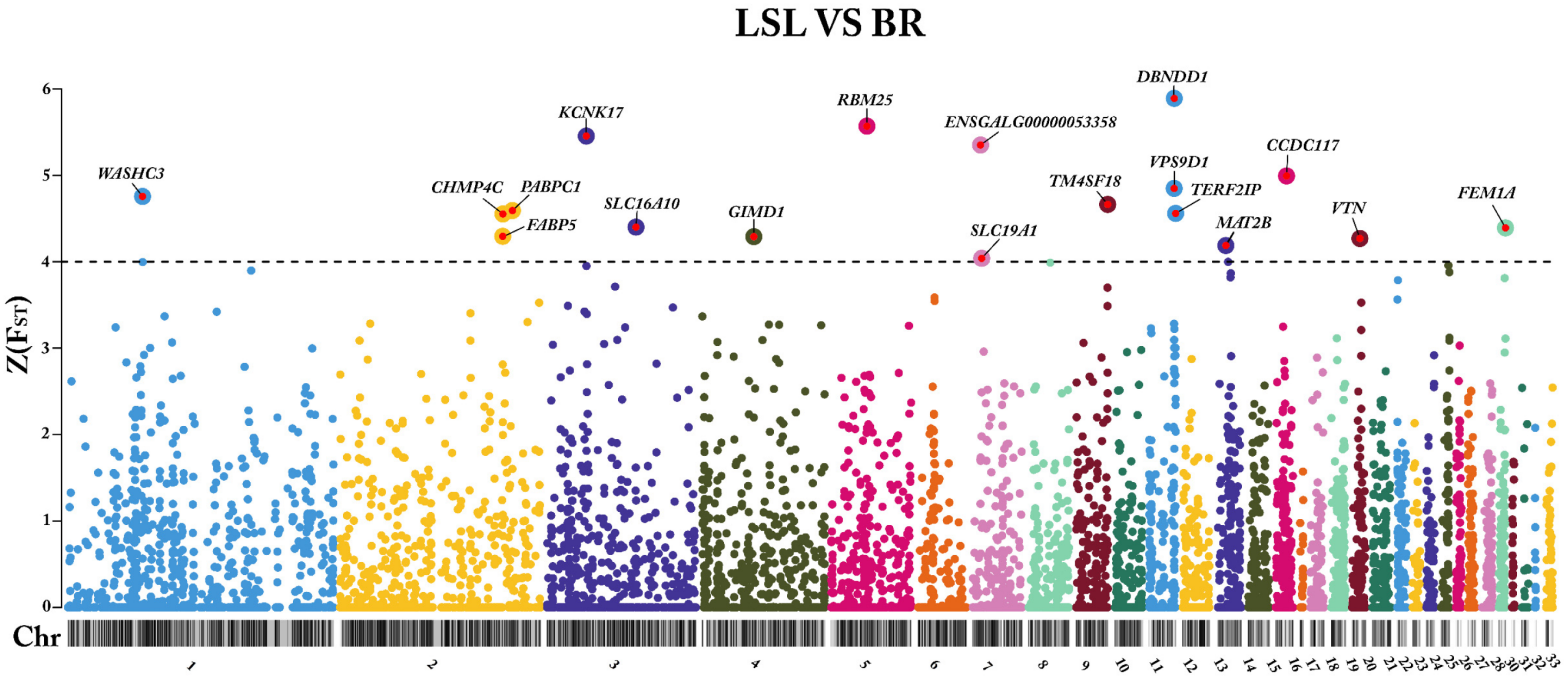
Detection of genetic differentiation between LSL and BR. Manhattan plot of gene‐specific *Z* (*F*_ST_) values comparing LSL and BR breeds. The black ideogram on the x‐axis shows the chromosomes, whereas the y‐axis indicates the *Z* (*F*_ST_) score for each gene. The black line indicates the cutoff threshold. Candidate genes that met the cut‐off criterion of *Z* (*F*_ST_) ≥ 4 are marked with gene symbols.

A total of 8974 genes represented by more than or equal to three SNPs were indicated to differentiate between LB and LSL. Out of these, 18 genes passed the cutoff criteria of *Z* (*F*_ST_) ≥ 4. These candidate genes were distributed on chromosomes: 1, 2, 3, 4, 5, 7,9, 11, 13, 15, 19, and 28. In the LSL vs. BR comparison, 3 genes including Dysbindin Domain Containing 1 (*DBNDD1*), VPS9 Domain Containing 1 (*VPS9D1*), and TERF2 Interacting Protein (*TERF2IP*) displayed the peak on chromosome 11 with *Z* (*F*_ST_) values ranging from 4.55 to 5.89. Similarly, 3 genes including Poly(A) Binding Protein Cytoplasmic 1 *(PABPC1*), Charged Multivesicular Body Protein 4C (*CHMP4C*), and Fatty Acid Binding Protein 5 (*FABP5*) were found on chromosome 2 with *Z* (*F*_ST_) values ranging from 4.29 to 4.59 (Figure 5). Interestingly, our results indicated that the highest *Z* (*F*_ST_) values were specified by genes on chromosome 5 (RNA‐Binding Motif Protein 25 (*RBM25*), *Z* (*F*_ST_) = 5.56), chromosome 3 (Potassium Two Pore Domain Channel Subfamily K Member 17 (*KCNK17*), *Z* (*F*_ST_) = 5.45), and chromosome 7 (*ENSGALG00000053358*, *Z* (*F*_ST_) = 5.35).

The remaining 9 candidate genes were located on chromosome 1 (WASH Complex Subunit 3 [*WASHC3*], *Z* (*F*_ST_) = 4.75), chromosome 3 (Solute Carrier Family 16 Member 10 [*SLC16A10*], *Z* (*F*_ST_) = 4.4), chromosome 4 (GIMAP Family P‐Loop NTPase Domain Containing 1 [*GIMD1*], *Z* (*F*_ST_) = 4.29), chromosome 7 (Solute Carrier Family 19 Member 1 [*SLC19A1*], *Z* (*F*_ST_) = 4.03), chromosome 9 (Transmembrane 4 L Six Family Member 18 [*TM4SF18*], *Z* (*F*_ST_) = 4.66), chromosome 13 (Methionine Adenosyltransferase 2B [*MAT2B*], *Z* (*F*_ST_) = 4.18), chromosome 15 (Coiled‐Coil Domain Containing 117 [*CCDC117*], *Z* (*F*_ST_) = 4.99), chromosome 19 (Vitronectin [*VTN*], *Z* (*F*_ST_) = 4.26), and chromosome 28 (Fem‐1 Homolog A [*FEM1A*], *Z* (*F*_ST_) = 4.39), as shown in Figure 5.

### 3.6 Colocalized QTL regions with F_ST_ genes

**FIGURE 6:**
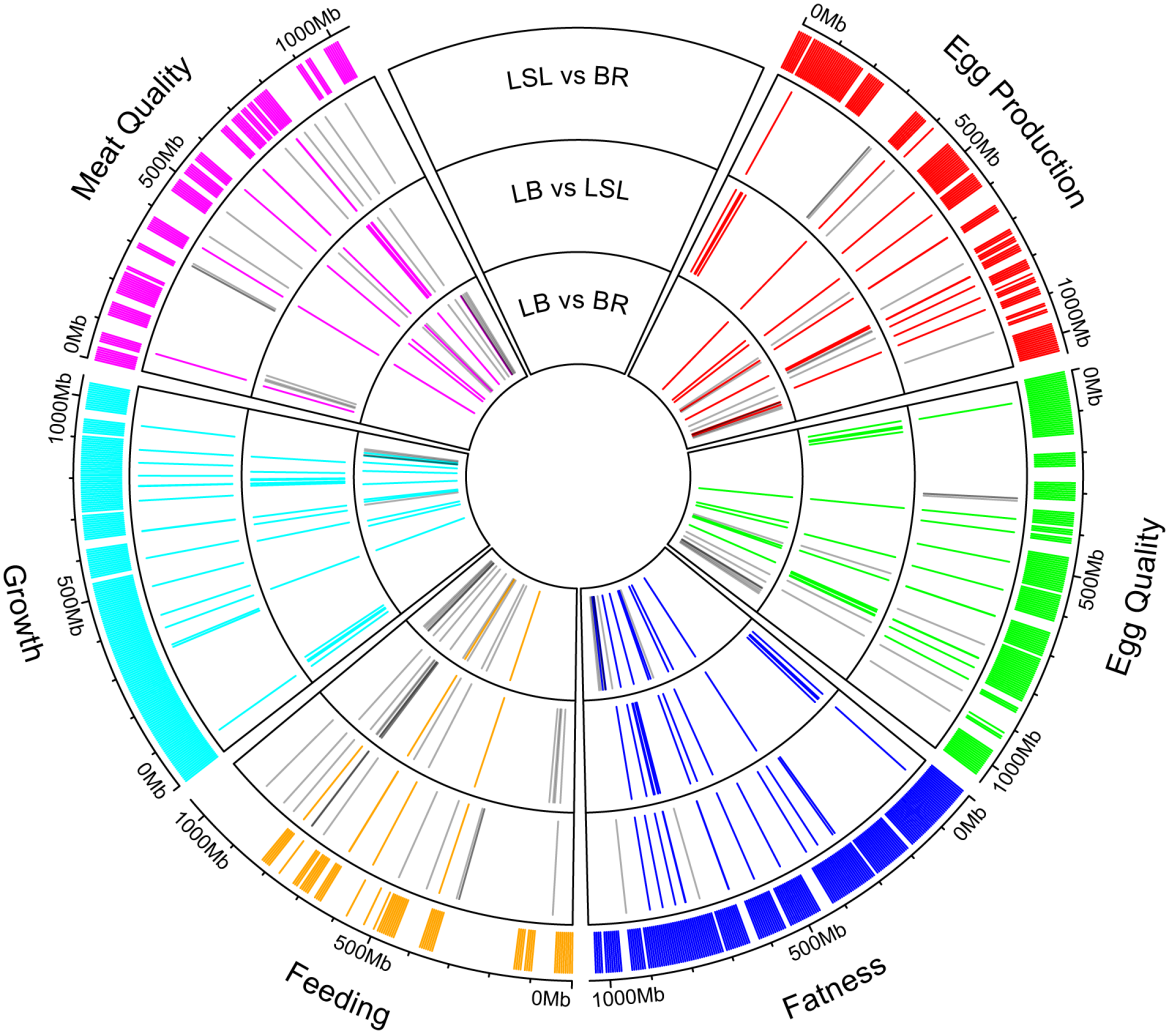
Candidate genes linked to chicken QTLs. Circosplot illustrates that genes selected as candidates in the pairwise F_ST_ analysis were assigned to a chicken QTL database. Each comparison was designated as layer 1: LB vs. BR; layer 2: LB vs. LSL; and layer 3: LSL vs. BR. The red, green, blue, orange, aquamarine, and pink lines in each layer indicate SNPs in genes meeting the cutoff criteria of *Z* (*F*_ST_) ≥ 4 (as shown in Figures 3, 4, and 5) and colocalized in QTL regions associated with egg production, egg quality, fat content, feeding, growth, and meat quality, respectively. The gray line indicates SNPs in genes that meet the cutoff criteria of *Z* (*F*_ST_) ≥ 4 but are not colocalized with any of the QTLs considered here. The outer layer represents the genomic location of the QTL regions.

The QTL database for chickens (https://www.animalgenome.org/cgi‐bin/QTLdb/GG/browse; chickenGRCg6a.bed) was used for mapping the *F*_ST_ region. Corresponding traits for laying or broiler chickens, including egg production, egg quality, fat content, growth, meat quality, and feeding categories were considered. A total of 7052 QTL regions were used, corresponding to egg production (668), egg quality (1465), fat content (284), growth (3142), meat quality (304), and feeding (1189). These QTL regions were shown in the outer layer of the cycle in Figure 6. We colocalized these QTL regions with the SNPs in genes meeting the cutoff criteria of *Z* (*F*_ST_) ≥ 4 when comparing LB with BR, LB with LSL, and LSL with BR. The candidate genes with *Z* (*F*_ST_) ≥ 4 were represented in a different layer of the cycle plot for each comparison. The exact location of the genes and the overlap of the QTL regions are shown in Table S2.

### 3.7 KEGG pathway enrichment analysis on top 1% of selected genes

**FIGURE 7:**
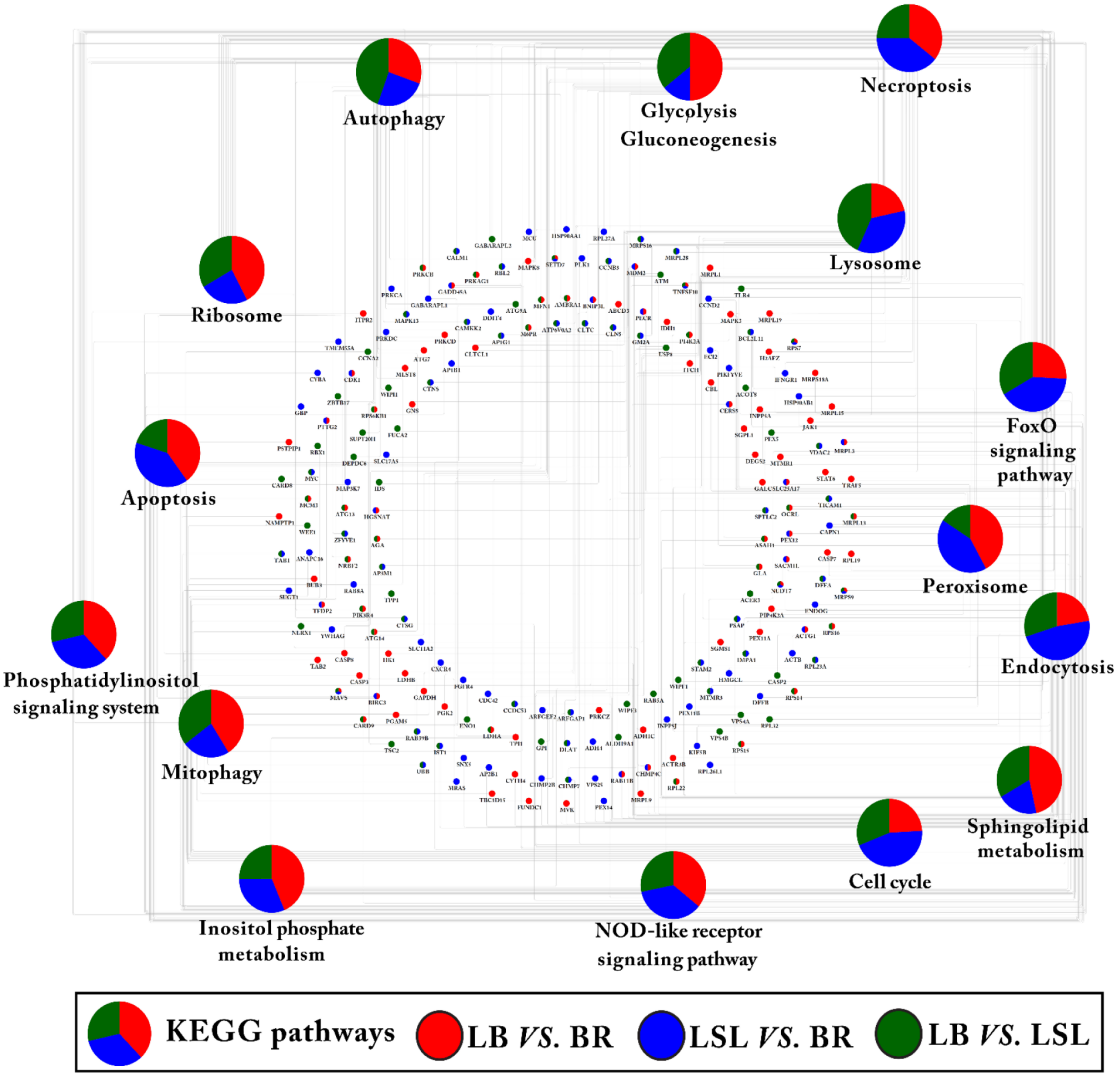
KEGG pathways enrichment analysis. The KEGG pathway enrichment analysis was performed on the selected genes with the top 1% of *Z* (*F*_ST_) values from all three comparison groups: (LB vs. BR, LSL vs. BR, and LB vs. LSL). The pie charts indicate the comparison group‐specific proportion of genes involved in the KEGG pathways. The red ellipse specifies the genes associated with LB vs. BR, the blue ellipse specifies the genes associated with LSL vs. BR, and the green ellipse specifies the genes from LB vs. LSL. KEGG pathways with *p* < 0.05 were significant.

All three comparison groups, (LB vs. BR, LSL vs. BR, and LB vs. LSL), were analyzed for KEGG pathway enrichment based on the 2697 selected genes with top 1% *Z* (*F*_ST_) values. Based on ClueGO enrichment analysis, the comparison groups LB vs. BR, LSL vs. BR, and LB vs. LSL were designated as clusters one, two, and three, respectively. Afterward, we used a term cluster for each comparison group. Among the genes with top 1% *Z* (*F*_ST_) values, cluster one had 906 genes, cluster two had 898 genes, and cluster three had 893 genes. A total of 15 KEGG pathways (Table S3), including cell cycle, peroxisome, FoxO signaling pathway, NOD‐like receptor signaling pathway, sphingolipid metabolism, necroptosis, ribosome, autophagy, endocytosis, mitophagy, glycolysis/gluconeogenesis, lysosome, inositol phosphate metabolism, phosphatidylinositol signaling system, and apoptosis were enriched by these selected genes with different proportions (Figure 7). The proportion of genes within the clusters and pathways were listed in Table 1.

**TABLE 1:** The top 1% of FST genes between chicken strains are enriched in KEGG pathways.

KEGG pathways	*p*‐value	Gene count	% genes cluster 1 (LB vs. BR)	% genes cluster 2 (LSL vs. BR)	% genes Cluster3 (LB vs. LSL)
Cell cycle	0.01	20	24.14	44.83	31.03
Peroxisome	0.01	14	42.11	42.11	15.79
FoxO signaling pathway	0.01	16	25.93	40.74	33.33
NOD‐like receptor signaling pathway	0.01	30	35.90	35.90	28.21
Sphingolipid metabolism	0.01	10	46.67	20.00	33.33
Necroptosis	0.02	21	35.71	39.29	25.00
Ribosome	0.02	20	42.42	24.24	33.33
Autophagy	0.02	26	30.56	25.00	44.44
Endocytosis	0.02	31	22.50	47.50	30.00
Mitophagy	0.03	13	41.18	23.53	35.29
Glycolysis/Gluconeogenesis	0.03	12	50.00	14.29	35.71
Lysosome	0.04	23	21.62	35.14	43.24
Inositol phosphate metabolism	0.04	11	43.75	31.25	25.00
Phosphatidylinositol signaling system	0.04	14	38.10	33.33	28.57
Apoptosis	0.04	18	40.00	40.00	20.00

### 3.8 Cis‐regulated genes in the jejunum mucosa of LB and LSL

Allele‐specific expression analysis was performed on 292,485 identified cis‐regulatory SNPs in jejunum mucosal transcripts of LB and LSL strains at five developmental stages (10, 16, 24, 30, and 60 weeks) to identify genes that are allele‐specifically regulated using the gene‐based ASEP approach. ASEP is based on a generalized linear mixed effects model with subject‐specific random effects to account for the correlation of multiple SNPs within the same gene (Fan et al., 2020). ASEP can detect ASE across multiple individuals, in our case in each strain and aging group.

**FIGURE 8:**
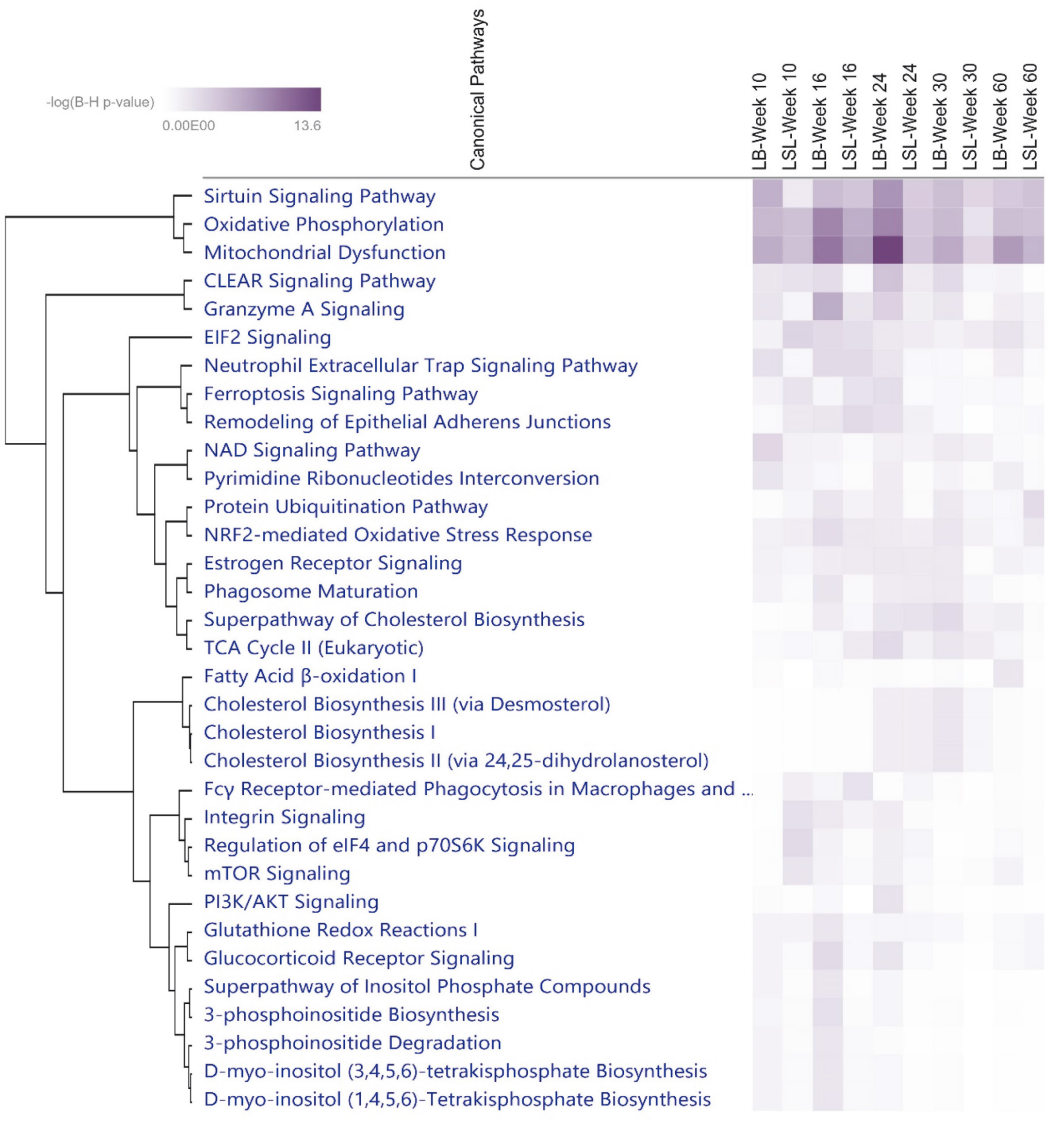
An IPA comparison analysis of the related canonical pathways of ASE genes per strain and time point. Related canonical pathways were hierarchically clustered and displayed with a heat map according to the negative logarithm of the Benjamini–Hochberg‐adjusted *p*‐value (B‐H).

Our results showed 4945 ASE genes with a *p*‐value of<0.01 (FDR < 5%) in at least one of the strains or aging groups (Table S4). A number of ASE genes identified within strains and time points, as well as the strain‐specific ASE genes, are listed in Table 2. A higher number of ASE genes was found in the LB strain than in the LSL strain at all time points (10, 16, 24, 30, and 60 weeks). We also found 7 ASE genes (FDR < 5%) common with fixation index analysis (*Z* (*F*_ST_) ≥ 4) in the comparison between LB and LSL, including *TERF*, *SAAL1*, *UBA32*, *C1H12ORF57*, *KCNK17*, *SLC13A2*, and *RAC2*. All ASE genes of each group with a *p*‐value of <0.01 (Table S4) were submitted for IPA pathway analysis and we found that most ASE genes are involved in energy metabolism, including Sirtuin Signaling Pathways, Oxidative phosphorylation, and mitochondrial dysfunction (Figure 8). A high number of ASE genes was found at the 24th and 30thweeks which is the peak of laying and these ASE genes were also enriched particularly in Cholesterol Biosynthesis. While the prelaying period (10 and 16 weeks), ASE genes were enriched in Inositol Phosphate Metabolism.

**TABLE 2:** A number of ASE genes were detected within the strains across all time points as well as strain‐specific ASE genes.

Strains	10 weeks	16 weeks	24 weeks	30 weeks	60 weeks	Total
LB	1295	1438	1773	1843	1708	4945
LSL	1099	1171	1469	1524	1276	
LB‐specific	110	151	193	184	219	857
LSL‐specific	59	135	158	178	161	691

**FIGURE 9:**
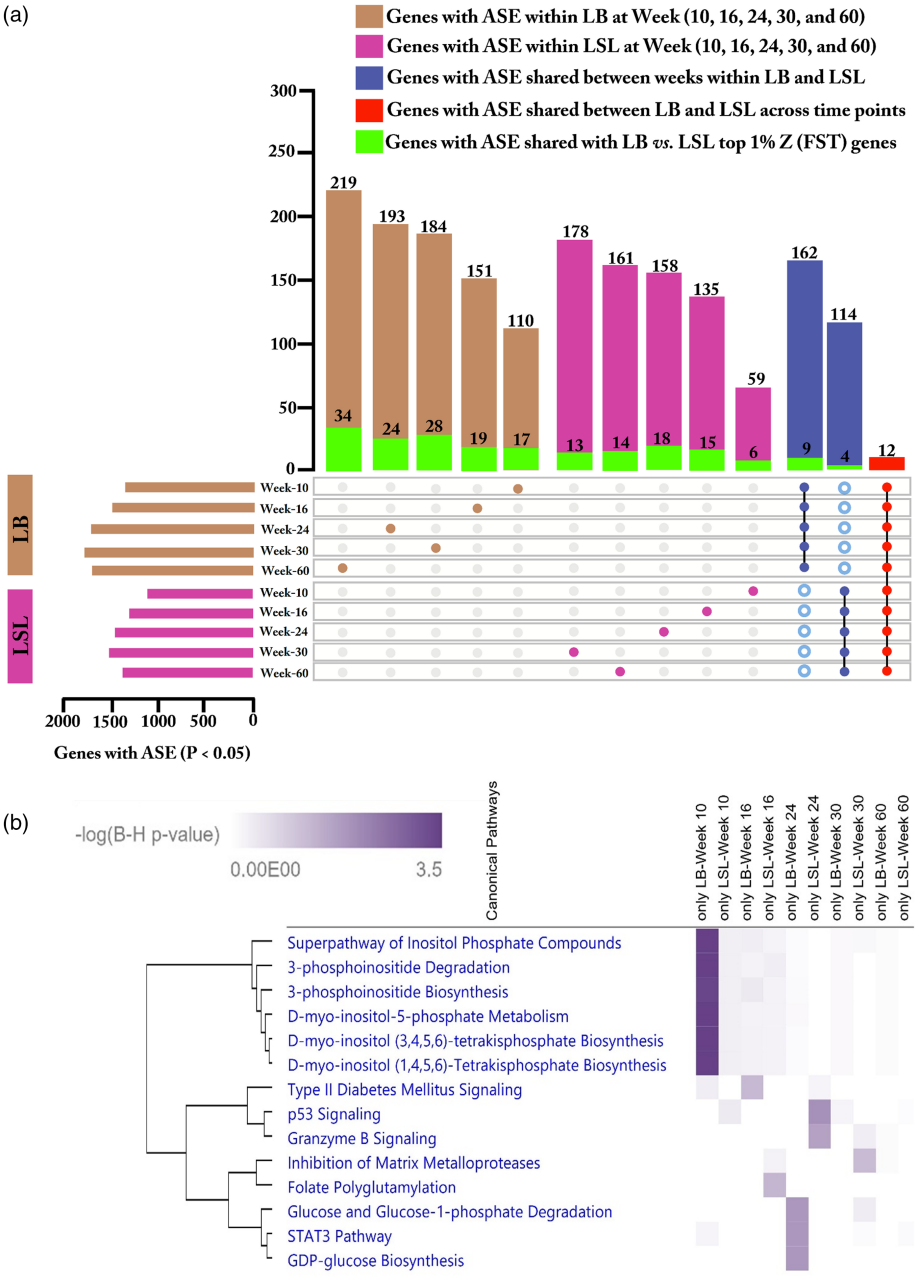
Allele‐specific expression within LB and LSL at different time points. (a) An upset plot indicates the number of genes with ASE within LB and LSL at weeks 10, 16, 24, 30, and 60. The brown bar and dots indicate the number of genes with ASE within the LB strain, whereas the pink bar and dots represent the number of genes with ASE within the LSL strain at five different ages (10, 16, 24, 30, and 60 weeks). The blue bar dots with the black line indicates the number of genes with ASE shared among the five time points within LB and LSL strains while the genes might also be present in LSL or LB at one of the time points (hollow blue dots). The red bar and dots with the black line illustrate the number of genes with ASE shared between LB and LSL across the five time points. The green bar indicates the number of genes shared between ASE analysis and the top 1% *Z* (*F*_ST_) genes from pairwise F_ST_ analysis of LB versus LSL strains. The horizontal brown and pink bars indicate the number of genes with ASE in LB and LSL with *p* < 0.05. (b) An IPA comparison analysis of the related canonical pathways of ASE genes specific for each strain and time point. Related canonical pathways were hierarchically clustered and displayed with a heat map according to the negative logarithm of the Benjamini–Hochberg‐adjusted *p*‐value (B‐H).

We also detected ASE genes that were strain‐ and age‐specific (Figure 9; Table S5). A total of 857 genes with a *p*‐value of <0.05 showed specific ASE in the LB strain of which 110, 151, 193, 184, and 219 showed ASE at weeks 10, 16, 24, 30, and 60, respectively. Similarly, 691 genes showed specific ASE in the LSL strain, of which 59, 135, 158, 178, and 161 showed ASE at weeks 10, 16, 24, 30, and 60, respectively. Our results showed that 162 genes with ASE in the LB strain were common across time, whereas 114 genes with ASE in the LSL strain were common between time points and weeks. Furthermore, we also identified 12 common genes that showed ASE across time points in both LB and LSL strains (*ARHGEF16, ENSGALG00000040002, ENSGALG00000048698, ENSGALG00000049751, NDUFA6, PAFAH2, PPA1, SLC37A4, SMIM20, TMEM51, TMSB15B*, and *TXN2*). Finally, we identified genes shared between ASE analysis and the top 1% *Z* (*F*_ST_) genes from pairwise F_ST_ analysis of LB versus LSL strains. In the LB strain, a total of 122 genes were common between ASE analysis and the top 1% *Z* (*F*_ST_) genes from pairwise F_ST_ analysis, including 17/110, 19/151, 24/193, 28/184, and 34/219 at weeks 10, 16, 24, 30 and 60, respectively. Similarly, in the LSL strain, a total of 66 genes were common between ASE analysis and the top 1% *Z* (*F*_ST_) genes from pairwise *F*_ST_ analysis, including 6/59, 15/135, 18/158, 13/178, and 14/161, respectively, at weeks 10, 16, 24, 30, and 60. Moreover, our result showed that 9/162 and 4/114 genes were common across weeks within strains LB and LSL, respectively, in the ASE analysis and the top 1% *Z* (*F*_ST_) genes from the pairwise F_ST_ analysis (Figure 9a). In addition, these strain‐ and age‐specific ASEs were subjected to IPA pathway analysis (Figure 9b). Interestingly, ASE genes which are specific only in LB laying strain at 10 weeks were enriched in Inositol Phosphate Metabolism (*DUSP15*, *IP6K1*, *OCRL*, *PDCD1*, *PDXP*, *PPP1R14D*, *PPP2R5D*, and *SET*). ASE genes in the LSL strain at 24thweek were enriched in p53 Signaling (*APAF1*, *CSNK1D*, *MDM4*, *PIK3C2A*, *PRKDC*, and *TP53BP2*) and Granzyme B Signaling (*APAF1*, *DFFA*, and *PRKDC*), whereas ASE genes from LB strain at 24thweek were enriched in Glucose and Glucose‐1‐phosphate Degradation (*HK1*, *HK2*, and *PGM2L1*) and STAT3 Pathway (*EGFR*, *IFNAR1*, *IL17RA*, *IL20RA*, *IL22RA1*, *IL4R*, and *PTPN6*).

## 4 DISCUSSION

During the last century, the success of the selective breeding of chickens specialized in laying eggs (layers) and fast‐growing meat birds (broilers) became apparent. Using whole‐genome sequence data, a recent study has elucidated selection forces in the genome of commercial chickens, particularly broilers and layers, compared with the ancestral red jungle fowl, and identified putative selection sweeps affecting loci likely to have been affected by the domestication and selection process associated with improved production (Qanbari et al., 2019). Our previous studies focused on the molecular and phenotypic characterization of the two laying hen strains LB and LSL. Interestingly, both lines have a similar performance on egg production but differ in body weight, immunity, bone metabolism, phytate degradation, and transcript abundance of genes that could be assigned to immune system processes and phosphorus metabolic processes (Habig et al., 2012, 2014; Omotoso et al., 2021; Ponsuksili et al., 2022; Sommerfeld, Omotoso, et al., 2020). Therefore, genetic variation between the two strains of laying hens (LB and LSL) and in contrast to the Cobb 500 broiler (BR) meat chickens was the subject of the present study. We used available RNA‐Seq data sets of the three chicken populations to derive reliable SNPs and genotypes for analyzing the genetic differentiation between strains and ASE of genes across different developmental stages of the laying hen.

### 4.1 Genes identified as candidates distinguishing between LB and LSL strains

By comparing LB vs. LSL, our pairwise fixation index analysis identified 17 genes that were selected as candidates and highly differentiated between the two‐layer strains (*Z* (*F*_ST_) ≥ 4). Several previous studies indicate that the immune and metabolic systems of both strains are acquired and maintained differently under high‐performance conditions (Iqbal et al., 2021, 2022; Ponsuksili et al., 2022). In line with these results, our study shortlisted the *CREBL2* on chromosome 1 as a candidate gene that was highly differentiated in the jejunum mucosa of LB and LSL strains with [*Z* (*F*_ST_) = 4.65]. In fact, CAMP‐responsive element‐binding protein‐like 2 (*CREBL2*) plays an important role in metabolic activities as a direct downstream target of *AMPK* (Altarejos & Montminy, 2011; Hu et al., 2021; Tiebe et al., 2019). A study on growth performance in New Hampshire and White Leghorn chicken lines identified a QTL region for body weight gain (Nassar et al., 2015). This QTL region harbors *CREBL2*, which was identified as a functional candidate in the present study. In addition, we identified five candidate genes on chromosome 11, including *TERF2*, *VPS9D1*, *PDP2*, *CMTM4*, and *PHLPP2*, which differed between the two strains. The candidate genes mentioned were involved in many biological processes. For example, TERF2 plays a key role in many processes during development and aging, including muscle and lipid metabolism (Robin et al., 2020). *TERF2* was also identified in this study as the ASE gene at the 24th week in LSL and is located in a QTL region for fatness and growth traits (Table S2). Consistent with a previous study that identified a QTL region on chromosome 11 associated with body weight traits (Jia et al., 2012). The other genes *VPS9D1* (GTPase activator activity and transporter activity), *PDP2* (pyruvate metabolism, Gray, 2014), CMTM4 (cytokine activity, Tan et al., 2022), and *PHLPP22* (protein regulating the cell's response to metabolic stress via the AMPK pathway, Yan et al., 2021) are mainly involved in immunological processes and energy metabolism, confirming previous findings that both strains use different strategies to maintain their immunity and metabolic activity under high‐performance conditions.

Furthermore, the gene SLC13A2 (Solute Carrier Family 13 Member 2), which codes for the sodium‐coupled citrate transporter (Akhtar et al., 2022) is worth mentioning here. Our recent study showed that *SLC13A2* is highly expressed in the intestinal mucosa, especially in the laying period (week 24), and was also identified as a biosignature associated with age and laying strain (LB vs. LSL; Ponsuksili et al., 2022). In the same study, we found that the expression of *SLC13A2* in the intestinal mucosa was strongly negatively correlated with phosphorus utilization (*r* = 0.73; Ponsuksili et al., 2022). Interestingly, based on the genetic variants, this study also selected *SLC13A2* as a candidate gene that differs between laying lines and as an ASE gene for the LB laying strain at the 24th and 30th week; we speculate that *SLC13A2* may be involved in the complex events that occur during the laying cycle and that it is strain specific during the selection process. This is consistent with a study showing that *SLC13A2* and *SLC35B4* are posttranscriptionally regulated in chickens via miRNAs, and the expression of SLC family genes associated with egg formation, oviposition, and embryonic development changes in chickens 3 and 20 h after ovulation (Lim et al., 2012). Interestingly, *SLC13A2* and *SLC35B4*, which were identified as functional candidate genes in this study, are located in the QTL region that has been associated with egg number (Atzmon et al., 2008) and egg production (Goto et al., 2011), respectively. The LB and LSL strains have different immunological phenotypes, with LB having an innate immunological phenotype, whereas LSL chickens showed an adaptive immunological phenotype (Hofmann et al., 2021). Interestingly, this study also identified *RAC2* on chromosome 1 as a functional candidate that is distinct between LB and LSL strains and as an ASE gene at week 16 of the LB strain. Consistent with this transcriptome analysis of the breast muscles of two native chickens with divergent feed efficiency revealed that Rac family small GTPase 2 (*RAC2*) is a critical signal transduction factor in immune cells and is associated with modulation of the various NADPH oxidase (NOXes) family members, responsible for the production of ROS in response to receptor activation such as inflammatory cytokines (Hordijk, 2006). *RAC2*‐deficient mice study shows pro‐inflammatory cytokines and chemokines were inhibited (Zou et al., 2017). In chicken, ROS are produced in phagosomes of chicken heterophils by *RAC2* to kill pathogens (Nambooppha et al., 2018). Together with the abundant evidence showing that RAC2 plays an immunomodulatory role, we proposed that this gene may be a factor responsible for the different immunological phenotypes of LB and LSL strains.

The role of Serum Amyloid A (*SAA*) family genes in the immune system of humans was well known, for instance, a variety of defense functions are induced by SAA, including neutrophil priming, leukocyte recruitment, and antiviral activity (Cai et al., 2007; Hatanaka et al., 2003; Lavie et al., 2006). Similarly, another study identified the *SAAL1* gene playing a significant role in the immune defense of *Oplegnathus fasciatus* fish species (Revathy et al., 2012). Interestingly, our fixation index analysis shortlisted the *SAAL1* as a functional candidate gene that was highly differentiated between the two strains [*Z* (*F*_ST_) = 4.98] and also was identified as an ASE gene at the 24th week of the LB strain. Therefore, we suggest that further research is required to deeply understand the role of the *SAAL1* gene in immunity and homeostasis in chickens. Catechol‐O‐Methyltransferase Domain Containing 1 (*COMTD1*) gene encodes for a methyltransferase with O‐methyltransferase activity (Nishioka et al., 2013). Previously, a comparative transcriptomics study between two‐layer chicken strains revealed that LSL hens had downregulated COMTD1, resulting in a reduced substrate conversion, and subsequently, LSL layers' humerus bone‐breaking strengths were lower than those of LB layers (Habig et al., 2012). In the present study, we also identified the *COMTD1* on chromosome 6 as a candidate gene providing additional evidence to the earlier finding.

*F*_ST_ analysis revealed several genes that differ between LB and LSL, some of which show ASE. These genes play a role in metabolic and immune pathways that have been shown to be recruited differently and show a phenotypic difference between the lines against a background of consistent high egg production of both lines.

### 4.2 Genes identified as candidates distinguishing between LB and BR strains

In the LB vs. BR comparison, our pairwise fixation index analysis identified 16 genes that were selected as candidates and highly differentiated between the LB and Cobb 500 broilers. According to a previous study, lipid metabolism and fat deposition processes significantly differed between broiler and layer chicken (Boschiero et al., 2018). Furthermore, during chicken embryogenesis, differences in lipid metabolism have been observed, for instance, broiler livers have higher triglyceride levels (Buzała et al., 2015). A study indicated that by activating CaM/CAMKK2/AMPK pathways, S100 Calcium Binding Protein A16 (*S100A16*) regulates lipid metabolism. In another study, the S100A16 gene was shown to promote adipogenesis in the mouse 3 T3‐L1 cell line (Liu et al., 2011). Interestingly, our study also identified *S100A16* as a candidate gene that was differentiated between the brown layer and broiler, we assume that this gene may be responsible for higher fat accumulation in the liver of the broiler.

Together with *S100A16*, we shortlisted two functional candidate genes: alpha‐1,2‐Mannosyltransferase (*ALG9/ENSGALG00000021174*) is involved in lipid‐linked oligosaccharide assembly and N‐Acylsphingosine Amidohydrolase‐1 (*ASAH1*) is involved in sphingolipid metabolism. These genes seem to play a vital role in lipid metabolism, but further studies in the chicken are required to explore their role in a broader spectrum. Interestingly, *ALG9* is also located in the QTL region for abdominal fat weight reported previously (Gao et al., 2009). A comparative study of QTL for body weight and growth in broiler and layer found a QTL region for body weight on chromosome 4 (Podisi et al., 2013). In our study, *ASAH1* was identified in this QTL region differing between layer and broiler lines. In addition, this QTL region also covers other fatness QTL, including abdominal fat percentage and abdominal fat weight. *ALG9* and *ASAH1* are associated with chicken growth and development in the present study, providing further evidence to support the previous finding. However, no fatness QTL was found colocalized with SNPs in *S100A16*. Previous research demonstrated that a higher expression of the deubiquitination gene Zinc Finger RANBP2‐Type Containing 1 (*ZRANB1*) boosted water‐holding capacity via enhancing protein stability (Huynh et al., 2013; Ponsuksili et al., 2010; Wu et al., 2020).

As a comparison, an earlier study found that broiler meat had a much higher average water‐holding capacity (13.50 ± 0.05%) than layer meat (11.82 ± 0.19%; Saleem et al., 2017). In accordance with these findings, the present study also identified the *ZRANB1* as a functional candidate gene on chromosome 6. However, no meat‐quality QTL was colocalized with *ZRANB1*.

Recently, a study reported that Gastric Inhibitory Polypeptide (*GIP*) plays a key role in bone size, growth, and development in chickens (Guo et al., 2020; Tan et al., 2021; Wang et al., 2020). Similarly, research has discovered that *GLP1* and *GIP* are related to synbiotic activity in broiler chickens (Seino et al., 2010). In growing broiler chickens, an injection of synbiotics could modify growth traits, immunological traits, and developmental traits on a broad spectrum (Madej et al., 2015; Madej & Bednarczyk, 2016). In this study, *GIP* and *GLP1R* were identified as candidate genes that differed strongly between BR and LB and were located within the body weight QTL regions on chromosome 27 (Kerje et al., 2003) and chromosome 3 (Carlborg et al., 2003), respectively. These QTL regions also overlapped with other QTL regions such as average daily gain, body weight, and body weight gain. We speculate that *GIP* and *GLP1R* are two essential functional candidates for studying differences in growth and immunological traits between broilers and laying hens.

A recent transcriptome study indicated the role of the Rac GTPase activating protein 1 (*RACGAP1*) gene in muscle growth and development by comparing two divergent broiler chicken groups, including a fast‐growth group and slow‐growth group and their results indicated that *RACGAP1* was differentially expressed between the two groups and involved in many biological processes related to muscle growth and development (Chen et al., 2019). In the present study, we shortlisted *RACGAP1* as a candidate gene that was differentiated between LB and BR and we propose that the *RACGAP1* gene may play a role in the faster muscle growth performance of the broiler compared with the layer type. A study indicated that PHD Finger Protein 20 (*PHF20*) gene positively regulates bone formation, further PHF20‐null mice showed a defective skeletal phenotype and complete knockout of *PHF20* indicated spinal bone defects (Yang et al., 2017). Interestingly, in this study, *PHF20* was found to be located in the QTL region for growth previously found on chromosome 20 (Wahlberg et al., 2009). Earlier, it has been shown that compared with layers and traditional lines, broiler tibiae were stronger, stiffer, and had lower stress values (Hocking et al., 2009). In this study, we identified *PHF20* as a candidate gene that was differentiated between LB and BR and we suggest further studies to investigate its role in the chicken osteoblast differentiation. In addition, candidate genes such as mitogen‐activated protein kinase 3 (MAPK3), which is involved in follicle development in chickens (Zhang et al., 2022), or the expression of HspB1, which prolongs lifespan and increases resistance to heat stress (Alexander et al., 2022), have also been identified as functional candidate genes that differ between LB and BR.

### 4.3 Genes identified as candidates distinguishing between LSL and BR strains

Comparing the LSL vs. BR, our pairwise fixation index analysis identified 18 genes that were selected as candidates and highly differentiated between the Lohmann Selected Leghorn (egg‐type) and Cobb 500 Broiler (meat‐type) chickens (Figure 5). We found three candidate genes *KCNK17*, *ENSGALG00000053358*, and *VPS9D1* that were common in comparisons between LSL vs BR and LB vs LSL and and were located in QTL regions for various traits. The eggshell formation is largely dependent on ion transport and Na+, Ca2+, and K+ ion channels are responsible for transporting Ca2+ from the plasma into the uterine lumen (Benos & Stanton, 1999). It has also been reported that *KCNK17* is involved in transmembrane ion transport (Li et al., 2019). Our study identified Potassium Two Pore Domain Channel Subfamily K Member 17 (*KCNK17*) as a functional candidate gene with ASE at the 30th week of the LB strain, and we assume that *KCNK17* might be involved in transporting Ca2+ from the plasma into the uterine lumen.

Previously, the yolk sac membrane expressed three thyroid hormone transporters, including *SLC16A2*, *SLC16A10*, and *SLCO1C1* during chicken embryonic development (Too et al., 2017). Most intriguingly, our study identified the *SLC16A10* as a functional candidate gene. Along with the well‐known thyroid hormone transporter *OATP1C1*, we propose this gene as a potential candidate for elucidating the role of thyroid hormone during embryonic development in chickens. The two folate transporters Solute Carrier Family 19 Member 1 (*SLC19A1*) and Solute Carrier Family 46 Member 1 (*SLC46A1*) were previously reported for their role in folate homeostasis in layer chicken (Jing et al., 2014). Folic acid supplementation has been reported to improve egg quality and immunity in laying hens. In the current study, we identified *SLC19A1* as a functional candidate gene that was well known for *folic acid transmembrane transporter activity*. The Poly(A) Binding Protein Cytoplasmic 1 (*PABPC1*) gene has been found to have a significant and positive correlation with breast muscle weight (Kang et al., 2021). Another study reported that increased *PABPC1* expression, as an active regulator of protein synthesis rates, in adult hearts increases the heart size and the ratio of heart to body weight (Chorghade et al., 2017). Similarly, our study also identified the *PABPC1* as a candidate gene and differentiated between layer and broiler, therefore, we speculate that *PABPC1* has a comparable impact on skeletal muscle growth and also indicates higher muscle growth in broiler than layer. *PABPC1* was also found in the QTL region of average daily gain and breast muscle weight.

A study demonstrated that compared with wild‐type mice, *FABP5* knockout mice demonstrated a 24% reduction in body fat mass and suggested that *FABP5* induced fat accumulation (Shibue et al., 2015). *FABP5* was found in QTL regions of fatness and growth, particularly, for average daily gain, body weight, breast muscle weight, and fat distribution traits. In the present study, we also identified *FABP5* as a candidate gene that was differentiated between white layer and broiler and highlighted the role of *FBAP5* in fat accumulation and body weight gain, we assume that this candidate gene may play a vital role in the higher growth rate in the broiler.

### 4.4 Allele‐specific expression (ASE) in the gut mucosa of laying strains

Allele‐specific expression (ASE) in the gut mucosa of two high‐yielding layer lines was used to determine cis‐regulatory divergence. In a previous study, it was shown that ASEP, a gene‐based method for ASE detection, can be used to summarize information across individuals at the population level and SNPs within the same genes using RNA‐seq data (Fan et al., 2020). ASE genes in each group were enriched in energy metabolism pathways, especially for the LB strain at the 24th week. It also emerges from this study that the LB strain not only has a higher body weight (Sommerfeld, Omotoso, et al., 2020) and a higher transcription level of genes (Omotoso et al., 2021; Ponsuksili et al., 2021) located in energy metabolism pathways but also that the genes in this pathway are ASE genes. In addition, ASE genes were found to be particularly enriched in cholesterol biosynthesis at the 24th and 30th weeks, the peak of the laying period, which is consistent with previous findings (Ponsuksili et al., 2022). Other interesting ASE genes identified in the prelaying period were enriched in inositol phosphate metabolism. All these apparent ASE genes point to specific biological processes related to the metabolic and nutritional requirements, especially energy and fat metabolism and phosphorus homeostasis, of egg production in both strains.

The enrichment analysis of ASE genes that were strain‐ and age‐specific (Figure 8) revealed their involvement in specific metabolic pathways. As shown in a previous study, egg‐laying activity alters the immune system toward a more pronounced humoral and innate immune response (Schmucker et al., 2021). On the one hand, cytotoxic T lymphocytes (CTL) and natural killer (NK) cells are important immune effectors related to granzyme B signaling, whereas tumor suppressor protein p53 (TP53) is an important transcriptional regulator that responds to a variety of cellular stresses and induces apoptosis or cell cycle arrest of damaged cells. ASE genes of LSL at 24 weeks were enriched in these signaling pathways. ASE genes in the LB 24‐week group were enriched in the STAT3 pathway, which deals with signaling messengers and transcription factors and is involved in normal cellular responses to cytokines and growth factors. This additional evidence of ASE genes in such pathways supports the previous finding that the immune response differs in LB hens and LSL hens and that the cellular arm of the immune system is more prominent in LB (Schmucker et al., 2021). In the LSL strain, we found *ALPL*, encoding the alkaline phosphatase enzyme and involved in bone mineralization, as an ASE gene. This result is consistent with previous studies reporting that the two strains of laying hens differed in body weight, immunity, bone metabolism, and phytate usage (Habig et al., 2012, 2014; Sommerfeld, Huber, et al., 2020; Sommerfeld, Omotoso, et al., 2020). In addition, higher ALP activity was found in the blood plasma of the LSL strain than in the LB strain in our previous study (Omotoso et al., 2021). Furthermore, at week 24, only the ASE genes within the LB strain were involved in the metabolic pathways, including glucose and glucose‐1‐phosphate degradation. Also, hexokinases (HK) belong to these pathways and catalyze the first step of glycolysis. HK2 is highly expressed in gut epithelium under microbial regulation via short‐chain fatty acid (SCFA) and is involved in immune responses and inflammation (Hinrichsen et al., 2021). We found *HK1* and *HK2* as specific ASE genes in the gut of LB at the 24thweek.

Interestingly, 7 of 17 genes with *Z* (*F*_ST_) ≥ 4 when comparing LB and LSL were ASE genes in at least one of the strain and age groups. For example, we found *KCNK17* and *SLC13A2* (FDR < 3 x 10^−6^) to be ASE genes at the laying peak (30 weeks) of the LB strain. As mentioned above, *KCNK17* and *SLC13A2* are involved in intestinal transport. These results suggest that the selection process is also associated with allelic imbalance at a particular time point of metabolic process and requirements, which is an essential step to decipher the genotype–phenotype map or functional diversity among chicken populations.

## 5 CONCLUSION

Our study provides several key candidate genes that were differentiated between three different chicken populations based on pairwise fixation index analysis. A number of these candidate genes are associated with characteristics such as growth, embryonic development, metabolism, immunology, and other traits of economic importance. Furthermore, Allele‐Specific Expression (ASE), in the gut mucosa of two high‐yielding layer chickens, suggests that the genetic architecture of different breeds may involve allelic heterogeneity, with multiple variants modifying the regulatory properties of different enhancers and regulating multiple genes and providing the baseline for further studies related to ASE for breed selection. Genetic differences between laying hen strains LSL and LB, as well as ASE patterns observed in the laying hen population in the current study, provide insight into the complex processes underlying selection in modern commercial breeding programs.

## CONFLICT OF INTEREST STATEMENT

The authors declare that they have no conflict of interest.

## Supporting information


Table S1

Table S2

Table S3

Table S4

Table S5

## Data Availability

Not applicable.
